# The Guidance of Verbal Working Memory Content to Attention

**DOI:** 10.3390/bs16060951

**Published:** 2026-06-09

**Authors:** Caibin Duan, Dequn Song, Ling Yuan, Lihua Zhang

**Affiliations:** 1 Students’ Affairs Division, Shenyang Agricultural University, Shenyang 110866, China; duancaibinhappy@syau.edu.cn (C.D.);; 2 College of Psychology, Liaoning Normal University, Dalian 116029, China

**Keywords:** verbal working memory, selective attention, visual search, memory match, guidance

## Abstract

This study used a dual-task paradigm to explore how stimuli with different semantic links to verbal working memory (WM) content affect attention across sensory channels and time courses. The results show: (1) In the visual channel, stimuli that were semantically the same as or similar to verbal WM content captured attention, with both types producing similar effects. This guidance weakened in the later stage of cognitive processing, possibly due to attentional control. (2) In the auditory channel, stimuli with same or similar semantics did not guide attention. These findings suggest that verbal WM content influences attention differently depending on sensory channel and processing time.

## 1. Introduction

People face large amounts of information every day, but only a small part is actually noticed and processed. Selective attention helps us focus on relevant information while ignoring distractions. It is shaped by bottom-up factors, such as salient stimuli that naturally draw attention (K. Jung et al., 2019), and top-down processes, such as attentional control that biases us toward task-relevant information (Han, 2017).

Working Memory (WM), as a top-down cognitive process, plays a guiding role in attention (Borders et al., 2022; Desimone & Duncan, 1995). When we actively hold information in WM, it can automatically direct attention to matching stimuli. This is known as the attentional guidance effect (Soto et al., 2008). According to the biased competition model (Desimone & Duncan, 1995), attentional resources are limited, and stimuli compete for processing. The content held in WM acts as an “attentional template” that boosts neural responses to matching features, giving those stimuli an advantage.

Many studies have used a dual-task paradigm to test this idea (Brockhoff et al., 2022; S. Jung et al., 2018; Wei & Zhou, 2020). Typically, participants first perform a memory task, then a visual search task in which they look for a target among distractors. One distractor matches the memory item. If that distractor captures attention before the target, it suggests that WM guides attention. For example, S. Jung et al. (2018) found that in real-world scenes, items matching WM content were more likely to attract attention. However, other studies found no such effect (Kirmsse et al., 2023; Woodman & Luck, 2007). This has led researchers to examine factors that influence whether WM content guides attention. Some studies suggest the effect depends on processing time (Fu et al., 2022; Han, 2017; Oberauer, 2019). In the early stages, visual WM easily guides attention; as the interval between stimuli (ISI) increases, this guidance fades. So, WM content does guide attention to some extent, but this is influenced by timing. Still, most studies have focused on visual WM using physical feature matching (e.g., shape or color). Few have looked at how verbal WM content guides attention, whether this depends on timing, or whether semantic matching plays a role.

Visual and verbal WM may guide attention differently. Visual WM research often relies on physical matches (Han, 2017; Yang et al., 2019). Verbal WM, in contrast, involves more abstract semantic matching (Pan, 2010). According to Baddeley’s four-component model (Hitch et al., 2019), visual WM mainly uses the visuospatial sketchpad, while verbal WM engages the phonological loop and episodic buffer. The episodic buffer can turn phonological information into cross-modal semantic representations. This may allow verbal WM to guide attention based on abstract categories (e.g., synonyms or same-category words) rather than physical features.

Findings on verbal WM guidance are mixed. Soto and Humphreys (2007) had participants memorize color or shape words, then search for a target among distractors. Distractors matching the memory item led to longer reaction times (RTs), suggesting automatic guidance. Malcolm et al. (2016) also found guidance using verbal information with real-world scenes.

But Calleja and Rich (2013) found the opposite. Participants memorized either an object image or its category name. Distractors could be exact matches, category matches (another object from the same category), or neutral. Attention was captured only when the distractor visually resembled the memory item. Category matching alone did not work. They argued that so-called “semantic guidance” might actually come from visual similarity.

Based on a detailed comparison of the two study designs, we propose that the following differences may explain the inconsistent findings. First, the memory materials differed: Soto and Humphreys (2007) used simple color words that easily evoke visual imagery, whereas Calleja and Rich (2013) used complex images and category names, which are more abstract. Second, the definition of semantic matching varied. In Soto and Humphreys, “semantic matching” essentially involved word–color matching (e.g., “red” with a red stimulus), which still relies on perceptual features. In Calleja and Rich, category matching used different exemplars of the same basic-level category (e.g., different cats), representing a purer semantic relationship. Thus, the effect in Soto and Humphreys may still depend partly on visual coding, while Calleja and Rich’s stricter design may have eliminated purely semantic effects. These findings are similar to existing research, indicating that factors such as the visual complexity of memory materials, the operational definition of semantic matching, and task duration are the main factors that affect whether the content of working memory can guide attention (Olivers et al., 2011; Soto et al., 2008).

Notably, Li et al. (2018) adopted a design that differs from the studies above and is highly relevant to the present work. In their study, the memory item was a visually presented white color word (e.g., “red”). The search task required locating a slanted line among differently colored shapes and judging its orientation. They systematically manipulated three matching relationships between distractors and the memory item: perceptual matching (distractor character identical to the memory word), semantic matching (distractor outline color matched the meaning of the memory word), and perceptual-semantic matching (both character and color matched). Each was compared with a no-match baseline. Results showed significantly longer response times (RTs) in all three matching conditions than in the baseline, indicating that verbal working memory content guides attention. More importantly, they were the first to compare the guidance effects of verbal WM content presented visually versus auditorily. They found a guidance effect in the visual condition but no such effect in the auditory condition.

In previous studies, the matching relationship between memory items and distractors has mostly been based on same semantics (i.e., the memory item and distractor share the same content, e.g., the word “yellow” with a yellow distractor). Pan (2010) extended this to abstract dimensions (i.e., the memory item and distractor belong to the same category, with the memory item as an abstract concept and the distractor as a concrete instance, e.g., the word “color” with a red distractor). He found that verbal WM still guides attention. However, no study has examined whether the guidance effects of verbal WM content differ between same-semantic and similar-semantic matching.

Thus, the existing literature leaves several questions unanswered. First, neither Soto and Humphreys (2007) nor Li et al. (2018) distinguished whether same-semantic (e.g., “red” with a red distractor) and similar-semantic (e.g., “red” with a yellow distractor) matching produce different guidance effects. Second, these studies did not systematically manipulate the ISI, leaving the time course of guidance unclear. Is guidance by verbal WM content, like that by visual WM content, also constrained by processing time? Third, although Li et al. (2018) compared visual and auditory channels, the auditory condition yielded no guidance effect, possibly due to cross-modal resource competition. However, no study has directly examined whether auditorily presented semantic matching stimuli can capture attention during the early stage of visual search.

To examine whether verbal working memory content guides attention, the present study used a dual-task paradigm with two closely timed tasks. First, participants performed a memory task in which they memorized a verbal item (a color word). Second, they completed a visual search task in which they quickly located a target while ignoring distractors. Critically, one distractor in the search display shared a semantic relationship (identical or similar) with the memorized content. Participants were explicitly informed that this distractor would never become the target. Therefore, if verbal working memory content automatically guides attention, this semantically matching distractor should capture attention and delay target responses. This logic is tested by comparing RTs between match conditions and a baseline condition (no semantic relationship).

Color words in this study (e.g., “red”) have a dual nature. They refer to a perceptual feature (the visual experience of redness) and also serve as a semantic proxy for the meaning of the memorized word (the concept of “red”). When the memory word is “red” and the distractor is a red arrow, the match involves both perceptual and semantic levels.

The present study distinguishes these two roles by comparing same-semantic (“red” with a red distractor) and similar-semantic (“red” with a yellow distractor) guidance. In the similar-semantic condition, the distractor color (yellow) does not match the memory word’s meaning (red). If the effect were purely driven by perceptual color matching, no guidance should occur in this condition. Therefore, equivalent effects in the two conditions would support semantic generalization rather than a purely perceptual account.

In summary: (1) It remains unclear whether verbal WM content can guide attention. (2) It is unknown whether such guidance is affected by processing time course. (3) It is unclear whether same- and similar-semantic matching produce equal degrees of attentional guidance. (4) Most previous studies used visual stimulus presentation, and less attention has been paid to auditory channels. The same information can be encoded through both visual and auditory channels, but the processing methods differ. Thus, it is unclear whether the influence of verbal WM content on attention is the same across sensory channels. Based on the above, the present study addressed three issues. First, by manipulating the semantic matching type (same semantics, similar semantics, baseline), we examined whether verbal WM content guides attention and whether the guidance effects of same- and similar-semantic matching are equivalent. Second, by manipulating ISI (250 ms vs. 950 ms), we examined the time course of attentional guidance. Third, we compared guidance effects between visual and auditory channels.

## 2. Experiment 1a: Effect of Verbal WM Content on Attention Under Visual Channel

### 2.1. Methods

Participants: A priori power analysis was conducted using G*Power 3.1 for a repeated-measures ANOVA with a medium effect size (f = 0.25), α = 0.05, and power (1 − β) = 0.80. This indicated a required sample size of 24. We recruited 38 undergraduate students (18 women) to account for potential exclusions. Their average age was 18.42 years. All students were right-handed, had normal or corrected-to-normal vision, normal hearing, and no color blindness or color weakness. The study was approved by the Ethics Committee of Shenyang Agriculture University, and written informed consent was obtained before the experiment. All methods mentioned in this research were performed in accordance with the relevant guidelines and regulations.

Stimuli: The experiment was programmed with E-Prime 2.0. Stimuli were displayed on a 15.6-inch color monitor with a screen resolution of 1024 × 768. Participants viewed the center of the screen from approximately 60 cm away. The experiment included memory items, detection items, and search items. The memory and detection items were color words: “red” and “yellow” (0.95° × 0.95°). The search items were arrows pointing up, down, left, or right (1.43° × 0.95°). One interfering arrow (i.e., distractor) was filled with red, yellow, or no fill color. The other arrows had no fill color.

### 2.2. Design and Procedure

The experiment used a 3 (Matching type: same semantics, similar semantics, baseline) × 2 (ISI: 250 ms, 950 ms) within-subject design. We manipulated the ISI between the memory task and the search task to dissociate cognitive processing stages. The 250 ms ISI represented the early stage, during which attentional control has not yet fully engaged. The 950 ms ISI represented the late stage, during which attentional control has sufficient time to intervene. Dependent variables were response times (RTs), search task accuracy, and memory task accuracy.

**Figure 1:**
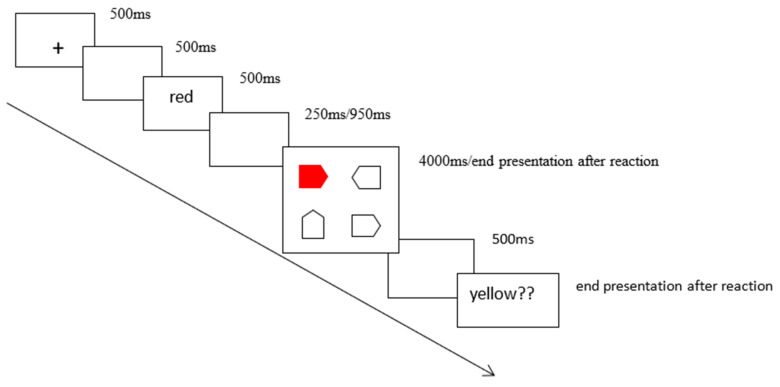
Example of trials with the same semantic matching type.

Each trial began with a fixation cross in the center of the screen for 500 ms. After a 500 ms blank screen, the memory task started. The memory item was presented for 500 ms. Participants were asked to remember the color word and read it aloud simultaneously. This ensured that the information was stored in WM (Cronin et al., 2020; Pan, 2010; Soto & Humphreys, 2007). Then, a blank screen was presented randomly for either 250 ms or 950 ms (50% each). After that, the search task began. The search display consisted of four arrows. Three arrows pointed left or right, and one of them was a distractor with a fill color. The remaining arrow pointed up or down and served as the target. Target and distractor positions were randomly assigned to four predefined screen locations (at the 2, 4, 8, and 10 o’clock positions on an imaginary circle with a radius of 3.65° visual angle from screen center). Each location was equally likely on each trial. Participants responded within 4000 ms by pressing the “L” key (up) or “M” key (down). After the response, a blank screen appeared for 500 ms, followed by the detection interface. Participants judged whether the detection item was the same as the memory item. They pressed “X” for same and “Z” for different (50% each). See Figure 1 for details.

Memory items and distractor fill colors formed three matching types: same semantics (word and fill color identical, e.g., “red” and a red arrow), similar semantics (word and fill color similar, e.g., “red” and a yellow arrow), and baseline (distractor had no fill color and no relation to the word). Each type accounted for 1/3 of trials. Participants were instructed to respond quickly and accurately in the search task. In the detection interface, they were asked to respond accurately without time pressure. Before the experiment, participants were clearly informed that distractors matching the WM content would never become the target. The experiment had three phases: (1) a practice phase of 16 trials to familiarize participants with the procedure, (2) a formal experiment of 96 trials divided into 2 blocks of 48 trials each, and (3) a rest phase in which participants could rest between blocks at their own discretion.

### 2.3. Results

Five participants with overall error rates above 20% were removed from analysis. This cutoff was chosen because the memory task involved a two-alternative forced choice (chance = 50%). An error rate above 20% suggests that the participant did not follow task instructions properly. Separate 3 (matching type) × 2 (ISI) repeated-measures ANOVAs were performed on detection task accuracy and search task accuracy (see Table 1). The results showed no significant main effects or interactions, all *Fs* < 2.97, all *ps* > 0.07.

**Figure 2:**
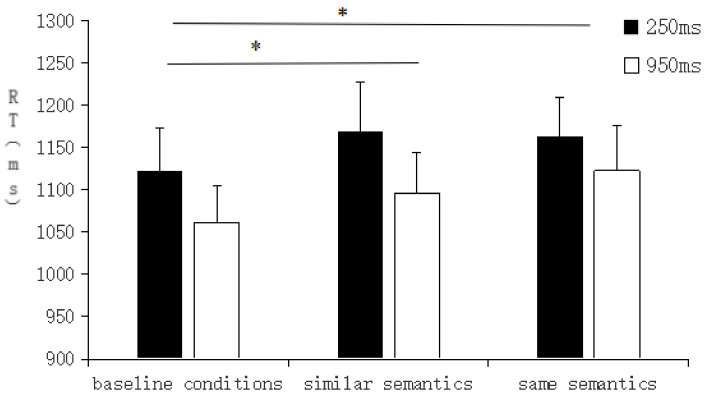
RT of search task under visual channel (error bars represent the standard errors of the mean, * *p* < 0.05).

Following conventional procedures (Bahle et al., 2018), we selected trials with correct responses in both memory and search tasks. We then excluded trials with RTs more than 2.5 standard deviations above or below the condition mean. A 3 × 2 repeated-measures ANOVA was conducted on search task RTs. The results showed a significant main effect of matching type, *F*(2, 64) = 3.38, *p* = 0.037, *η_p_*^2^ = 0.095. Post hoc multiple comparisons using LSD indicated that RTs under same- and similar-semantic conditions were significantly slower than under baseline (both *ps* < 0.04). There was no significant difference between same- and similar-semantic conditions (*p* > 0.05). The main effect of ISI was significant, *F*(1, 32) = 16.33, *p* < 0.001, *η_p_*^2^ = 0.341. RTs at 250 ms were significantly slower than at 950 ms. The interaction was not significant, *F*(2, 64) = 0.43, *p* > 0.05. See Figure 2.

### 2.4. Discussion

Experiment 1a showed that search RTs were longer in same- and similar-semantic conditions than in baseline. This finding confirms that verbal working memory content guides attention under the visual channel, consistent with previous studies. For example, Pan (2010) found that abstract semantic categories (e.g., the word “color” with a red distractor) guided attention. Sun et al. (2015) reported that conceptually generated working memory templates (e.g., the word “rose” with a red distractor) captured attention even without physical matching.

Notably, the effect sizes did not differ between the two matching conditions. This suggests semantic generalization: semantically similar features within the same category also capture attention. However, an alternative explanation is that the effect was due to the novelty of the colored stimulus rather than semantic guidance. Experiment 1b was designed to test this possibility.

## 3. Experiment 1b: The Role of Stimulus Novelty on Attention in the Absence of a Memory Requirement

Color novelty refers to the physical salience of a colored stimulus when it appears in the search display—specifically, the fact that colored stimuli are more perceptually distinct and attention-grabbing than uncolored (baseline) stimuli. Experiment 1b tested whether the RT slowing in Experiment 1a was due to this color novelty itself rather than to semantic matching. Participants were instructed to ‘just watch’ the color word without memorizing it, then performed the same search task. If the same RT slowing had occurred in Experiment 1b, novelty would have explained Experiment 1a; the fact that the effect disappeared indicates that the effect in Experiment 1a reflects guidance by working memory content, not simply physical salience.

### 3.1. Methods

Participants: 32 undergraduate students (19 women) were selected to participate in the experiment, with an average age of 18.78 years. All were right-handed, had normal or corrected-to-normal vision, normal hearing, and no color blindness or color weakness. The study was approved by the Ethics Committee of Shenyang Agriculture University. Written informed consent was obtained. All methods followed relevant guidelines.

Stimuli: Same as Experiment 1a.

### 3.2. Design and Procedure

The design was the same as Experiment 1a. The procedure was the same except that, during memory item presentation, participants were told they did not need to remember the word and should just watch it. No detection interface was presented.

### 3.3. Results

3 participants with overall error rates above 20% were removed. Separate 3 × 2 repeated-measures ANOVAs on search task accuracy (see Table 2) showed no significant main effects or interactions (all *Fs* < 0.44, all *ps* > 0.58).

**Figure 3:**
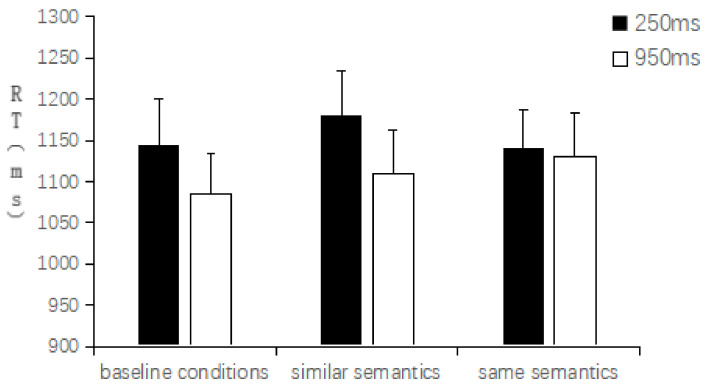
RT of search task under visual channel (error bars represent the standard errors of the mean).

Using the same RT trimming procedure as in Experiment 1a, a 3 × 2 repeated-measures ANOVA on RTs showed a significant main effect of ISI, *F*(1, 28) = 6.85, *p* = 0.014, *η_p_*^2^ = 0.196. RTs at 250 ms were slower than at 950 ms. The main effect of matching type was not significant, *F*(2, 56) = 1.39, *p* > 0.05. The interaction was not significant, *F*(2, 56) = 1.46, *p* > 0.05. See Figure 3.

### 3.4. Discussion

Experiment 1b revealed no main effect of matching type. This indicates that the novelty of the colored stimulus alone is insufficient to produce attentional capture. Therefore, the RT slowing observed in Experiment 1a reflects semantic guidance by verbal working memory content, not stimulus novelty.

Together, Experiments 1a and 1b demonstrate that under the visual channel, verbal working memory content reliably guides attention and that this effect exhibits semantic generalization. Experiment 2 next examines whether a similar effect occurs in the auditory channel.

## 4. Experiment 2: Effect of Verbal WM Content on Attention Under Auditory Channel

### 4.1. Methods

Participants: 35 undergraduate students (20 women) participated. Their average age was 18.33 years. All were right-handed, had normal or corrected-to-normal vision, normal hearing, and no color blindness or color weakness. The study was approved by the Ethics Committee of Shenyang Agriculture University. Written informed consent was obtained. All methods followed relevant guidelines.

Stimuli: Except that the memory items and the detection items were audio files, the other experimental materials were the same as Experiment 1a. The audio content was “red” or “yellow” spoken by a female voice, with a duration of less than 500 ms. The audio files used two channels, a sampling rate of 44.1 kHz, and an intensity of 66 dB.

### 4.2. Design and Procedure

The design was the same as Experiment 1a. The procedure was the same, except that the memory items were presented auditorily. A blank screen was presented for 500 ms before the search task. Detection items were also presented auditorily, with a blank screen until the participant responded.

### 4.3. Results

8 participants with overall error rates above 20% were removed. This relatively high exclusion rate may be due to the higher difficulty of the auditory memory task and some participants failing to maintain articulatory suppression. Separate 3 × 2 repeated-measures ANOVAs on memory and search task accuracy (see Table 3) showed no significant main effects or interactions (all *Fs* < 2.50, all *ps* > 0.11).

**Figure 4:**
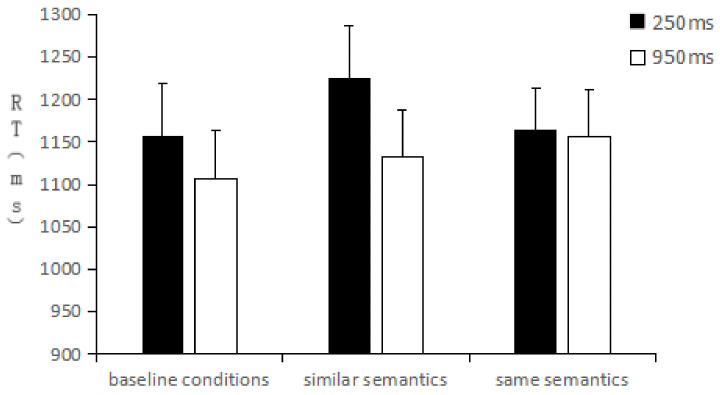
RT of search task under auditory channel (error bars represent the standard errors of the mean).

Using the same RT trimming procedure, a 3 × 2 repeated-measures ANOVA on RTs showed a significant main effect of ISI, *F*(1, 26) = 6.59, *p* = 0.018, *η_p_*^2^ = 0.211. RTs at 250 ms were slower than at 950 ms. The main effect of matching type was not significant, *F*(1.53, 39.75) = 1.87, *p* > 0.05. The interaction was not significant, *F*(2, 52) = 1.74, *p* > 0.05. See Figure 4.

### 4.4. Discussion

Experiment 2 revealed no significant main effect of matching type in the auditory channel. Thus, verbal working memory content did not guide visual attention when presented auditorily. This result contrasts with the visual-channel findings.

Several explanations may account for this absence of guidance.

First, resource competition: In our dual-task paradigm, the auditory memory task and visual search task competed for limited attentional resources. The visual search task was relatively more difficult and required greater resources, thus dominating the competition.

Second, active suppression: During a difficult visual search task, individuals may actively suppress task-irrelevant information from other sensory modalities (e.g., audition) to avoid cross-modal interference. This suppression may weaken any potential guidance from auditory memory content.

Third, modality-specific encoding: Auditory verbal information may be encoded primarily in a phonological form (Baddeley, 2012). Compared with visually presented words, this phonological code may be less effective at activating the abstract semantic representations needed to guide visual attention.

These explanations are not mutually exclusive. A non-significant result does not equate to evidence of no effect. Future studies with larger samples are needed to disentangle these possibilities.

## 5. General Discussion

This study explored how verbal working memory content guides attention across different sensory channels. In Experiment 1a, we found that compared with the baseline condition, search RTs were slower under both same- and similar-semantic conditions. One interpretation is that under the visual channel, stimuli with the same or similar semantics as verbal WM content guide attention. This supports the biased competition model. Such stimuli are more likely to capture attention, interfere with visual search, and slow responses (Li et al., 2018). Another possibility is that this effect is related to stimulus novelty. In the search task, the same- and similar-semantic conditions included colored stimuli, whereas the baseline did not. Colored stimuli are more salient and novel, which might attract attention regardless of WM content. Thus, individuals might first attend to the novel colored stimulus and then to the target, resulting in slower RTs.

However, Experiment 1b found no significant difference in search RTs among the three conditions when participants did not have to memorize the word. This result aligns with previous studies (Olivers et al., 2006). It indicates that novel stimuli alone do not guide attention in this task. Similarly, Experiment 2 found no significant difference in search RTs among conditions in the auditory channel. The results of Experiment 2 differed from those of Experiment 1a when facing novel stimuli. Therefore, the slower RTs in same- and similar-semantic conditions compared with baseline indicate that under the visual channel, stimuli with the same or similar semantics as verbal WM content guide attention. This effect is not simply due to stimulus novelty.

Furthermore, even though participants were clearly informed before the experiment that the stimulus matching the memory item would never become the target, that stimulus still captured attention preferentially. This finding is consistent with many studies (Han, 2017; Kawashima & Matsumoto, 2017). It indicates that the guidance of verbal working memory content to attention is not only preferential but may also be automatic.

Experiment 1a also found no significant difference in search RTs between same- and similar-semantic conditions. This suggests that verbal WM can guide attention based on either same- or similar-semantic matching (Chen & Du, 2017; Sasin & Nieuwenstein, 2016). Moreover, the guidance effects of the two matching types are equivalent. In contrast, visual working memory guides attention only based on exact, same-semantic matching (Han, 2017; Yang et al., 2019). This pattern differs from that of verbal working memory.

This difference may be related to Baddeley’s four-component model of working memory (Hitch et al., 2019). The model includes the central executive, visuospatial sketchpad, phonological loop, and episodic buffer. The visuospatial sketchpad encodes visual information. The phonological loop encodes phonological information. The episodic buffer converts and processes multi-channel information. Although verbal WM primarily encodes verbal information, it may be recoded into visual or other semantic information after passing through the episodic buffer. This enables attentional guidance based on abstract dimensional information. Such recoding relies on semantic associations and involves brain regions such as the left inferior frontal gyrus and left occipitotemporal cortex (Soto et al., 2012). In attentional guidance by verbal WM, both the phonological loop and episodic buffer are involved. In visual WM research, articulatory suppression is often used to prevent verbal coding, ensuring that only visual coding occurs. In the four-component model, only the visuospatial sketchpad is involved in visual WM. Therefore, visual WM content guides attention only based on exact, same-semantic matching.

In addition, we found significant differences in search RTs between ISIs. Compared with the early stage (250 ms), individuals showed significantly faster search RTs in the late stage (950 ms). This indicates that the guiding effect of verbal WM content on attention gradually weakens in the late stage. This may be related to attentional control. In search tasks, there is both guidance from verbal WM content and inhibition of irrelevant stimuli by attentional control. These two processes constrain each other (K. Jung et al., 2019). In the early stage, attentional control is relatively weak, and attention is mainly guided by verbal WM content. In the late stage, attentional control begins to exert influence. The stronger the attentional control, the better individuals can suppress irrelevant stimuli. Some studies have even found that under strong attentional control, stimuli matching WM are not guided but actively suppressed (Hu & Zhang, 2016). In this study, verbal WM content was task-irrelevant information during search. In the late stage, participants suppressed this stimulus through attentional control. Moreover, the frequency of distractors matching WM was not high, so participants’ motivation for attentional control was not strong. Therefore, search RTs were faster in the late stage, but the guiding effect of verbal WM on attention still remained.

The findings of the present study are consistent with and extend those of Li et al. (2018). Both studies confirmed that semantically matching distractors reliably prolong search RTs in the visual channel, demonstrating stable attentional guidance by verbal working memory. Building on this consistency, the present study revealed two novel findings that were not examined by Li et al. First, we found semantic generalization. By comparing same- and similar-semantic matching (e.g., “red” with a yellow distractor), we found no significant difference in effect sizes. This indicates that guidance spreads to semantically similar features within the same category, supporting the role of the episodic buffer in semantic representation (Hitch et al., 2019). Second, we examined time course. Li et al. did not manipulate ISI. By setting ISIs of 250 ms and 950 ms, we found that the guidance effect was robust at the early stage but weakened at the late stage. This suggests that attentional control gradually engages over time.

A critical concern is whether our findings reflect semantic processing or simply attentional capture by colored stimuli. Experiment 1a alone cannot distinguish between these accounts, because both same- and similar-semantic conditions involved colored distractors and produced equivalent effects.

Experiment 1b addressed this concern by removing the memory requirement. Under passive viewing (no memory task), colored distractors produced no guidance effect. This shows that color salience alone is insufficient to guide attention; an active working memory representation is necessary.

Thus, while colored distractors are visually salient, the results of Experiment 1b rule out a pure perceptual account. The guidance effects observed in Experiment 1a can be attributed to semantic matching, not to color novelty or physical salience.

Admittedly, complete separation of color-based and meaning-based attention is challenging with the present stimulus set, which conflates color perception and color meaning. Nevertheless, our comparison between same- and similar-semantic matching holds perceptual properties constant, allowing us to isolate the semantic contribution within the current design.

Experiment 2 revealed that when verbal WM content was presented auditorily, the main effect of matching type was not significant, indicating no attentional guidance effect. This result contrasts with the visual-channel findings of Experiment 1a. One possible explanation is resource competition. In our dual-task paradigm, the memory task (auditory) and search task (visual) occurred concurrently, competing for limited attentional resources. The search task (quickly locating a target arrow among multiple distractors) was relatively more difficult and required greater attentional resources. Thus, it dominated resource competition and received a larger share of attentional resources. The auditory memory task received fewer resources, making it difficult to adequately maintain and process its content. When auditory memory content is under-resourced, its semantic relationship with distractors in the search task cannot be effectively detected, thereby failing to produce an attentional guidance effect. Moreover, during a difficult visual search task, individuals may actively suppress task-irrelevant information from other sensory modalities (e.g., the auditory modality) to avoid interference. This suppression may further weaken the guidance of visual attention by auditorily presented verbal working memory content.

Experiment 2 also found significant differences in search RTs between ISIs. Compared with the early stage (250 ms), individuals had faster search RTs in the late stage (950 ms). This indicates that attentional control also plays a role in the auditory channel.

## 6. Limitations

First, although the a priori power analysis indicated a required sample size of 24, the final sample sizes after exclusions (33, 29, and 27) were relatively modest. This increases the risk of Type II error. The null effect in Experiment 2 may be partly due to this. A non-significant result does not equal evidence of no effect. Second, we used repeated-measures ANOVA to analyze RT data, but RT data often violate normality assumptions, and ANOVA is sensitive to such violations (Lo & Andrews, 2015). Linear mixed models are more robust. Future studies should re-analyze the data using such models. Third, although we instructed participants to read the memory word aloud to encourage phonological encoding, color words such as “red” and “yellow” may still evoke visual imagery. Thus, we cannot completely rule out visual recoding. Future studies could use abstract words without visual imagery (e.g., “justice,” “truth”) as memory materials.

## 7. Conclusions

Under the visual channel, stimuli with the same or similar semantics as verbal WM content guide attention, and the two types of guidance are equally strong. This guidance effect gradually weakens in the late stage of cognitive processing, which may be related to attentional control.

Under the auditory channel, stimuli with the same or similar semantics as verbal WM content do not guide attention.

## Figures and Tables

**Table 1:** Accuracy rates under different conditions ($\bar{x}$ ± *s*, %, *n* = 33).

ISI	Accuracy Rates of Memory Task			Accuracy Rates of Search Task		
	Same Semantics	Similar Semantics	Baseline Conditions	Same Semantics	Similar Semantics	Baseline Conditions
250 ms	0.95 ± 0.06	0.91 ± 0.11	0.93 ± 0.08	0.98 ± 0.04	0.98 ± 0.03	0.98 ± 0.05
950 ms	0.96 ± 0.06	0.92 ± 0.09	0.95 ± 0.08	0.98 ± 0.04	0.99 ± 0.03	0.98 ± 0.04

**Table 2:** Accuracy rates under different conditions ($\bar{x}$ ± *s*, %, *n* = 29).

ISI	Accuracy Rates of Search Task		
	Same Semantics	Similar Semantics	Baseline Conditions
250 ms	0.96 ± 0.11	0.96 ± 0.12	0.97 ± 0.07
950 ms	0.96 ± 0.09	0.96 ± 0.10	0.97 ± 0.07

**Table 3:** Accuracy rates under different conditions ($\bar{x}$ ± *s*, %, *n* = 27).

ISI	Accuracy Rates of Memory Task			Accuracy Rates of Search Task		
	Same Semantics	Similar Semantics	Baseline Conditions	Same Semantics	Similar Semantics	Baseline Conditions
250 ms	0.92 ± 0.09	0.92 ± 0.11	0.94 ± 0.09	0.95 ± 0.12	0.96 ± 0.12	0.97 ± 0.07
950 ms	0.96 ± 0.06	0.93 ± 0.10	0.95 ± 0.07	0.97 ± 0.09	0.96 ± 0.11	0.99 ± 0.04

## Data Availability

The data and materials used in this study are available upon request from the corresponding authors.
